# Computational model of tranexamic acid on urokinase mediated fibrinolysis

**DOI:** 10.1371/journal.pone.0233640

**Published:** 2020-05-26

**Authors:** Tie Bo Wu, Thomas Orfeo, Hunter B. Moore, Joshua J. Sumislawski, Mitchell J. Cohen, Linda R. Petzold

**Affiliations:** 1 Department of Mechanical Engineering, University of California Santa Barbara, Santa Barbara, California, United States of America; 2 Department of Biochemistry, University of Vermont, Burlington, Vermont, United States of America; 3 Department of Surgery, Denver Health and Hospital Authority, Denver, Colorado, United States of America; Medical College of Georgia, Augusta, UNITED STATES

## Abstract

Understanding the coagulation process is critical to developing treatments for trauma and coagulopathies. Clinical studies on tranexamic acid (TXA) have resulted in mixed reports on its efficacy in improving outcomes in trauma patients. The largest study, CRASH-2, reported that TXA improved outcomes in patients who received treatment prior to 3 hours after the injury, but worsened outcomes in patients who received treatment after 3 hours. No consensus has been reached about the mechanism behind the duality of these results. In this paper we use a computational model for coagulation and fibrinolysis to propose that deficiencies or depletions of key anti-fibrinolytic proteins, specifically antiplasmin, a1-antitrypsin and a2-macroglobulin, can lead to worsened outcomes through urokinase-mediated hyperfibrinolysis.

## Introduction

Severe trauma often induces a coagulopathic state known as Acute Traumatic Coagulopathy (ATC) that manifests in increased bleeding and resultant mortality [1]. The mechanisms underlying ATC are not yet fully understood, making treatment difficult [2]. One treatment in particular, tranexamic acid (TXA), has been found to be often effective [3, 4]. However, there are unknown circumstances in which the treatment has been reported to worsen the condition, with increased bleeding and mortality. A meta-analysis of the CRASH-2 study found that the relative risk of death due to bleeding in patients who received TXA compared to placebo was 0.68 for patients who arrived within 1 hour after injury, 0.79 for patients who arrived between 1 and 3 hours after injury, but 1.44 for patients who arrived more than 3 hours after injury [5, 6]. The risk of worsening the situation is one of the main reasons why many health care organizations are hesitant to incorporate TXA into their trauma protocol. Developing a mechanistic understanding of how TXA can produce such contrasting results is necessary for widespread adoption of the treatment.

**Fig 1:**
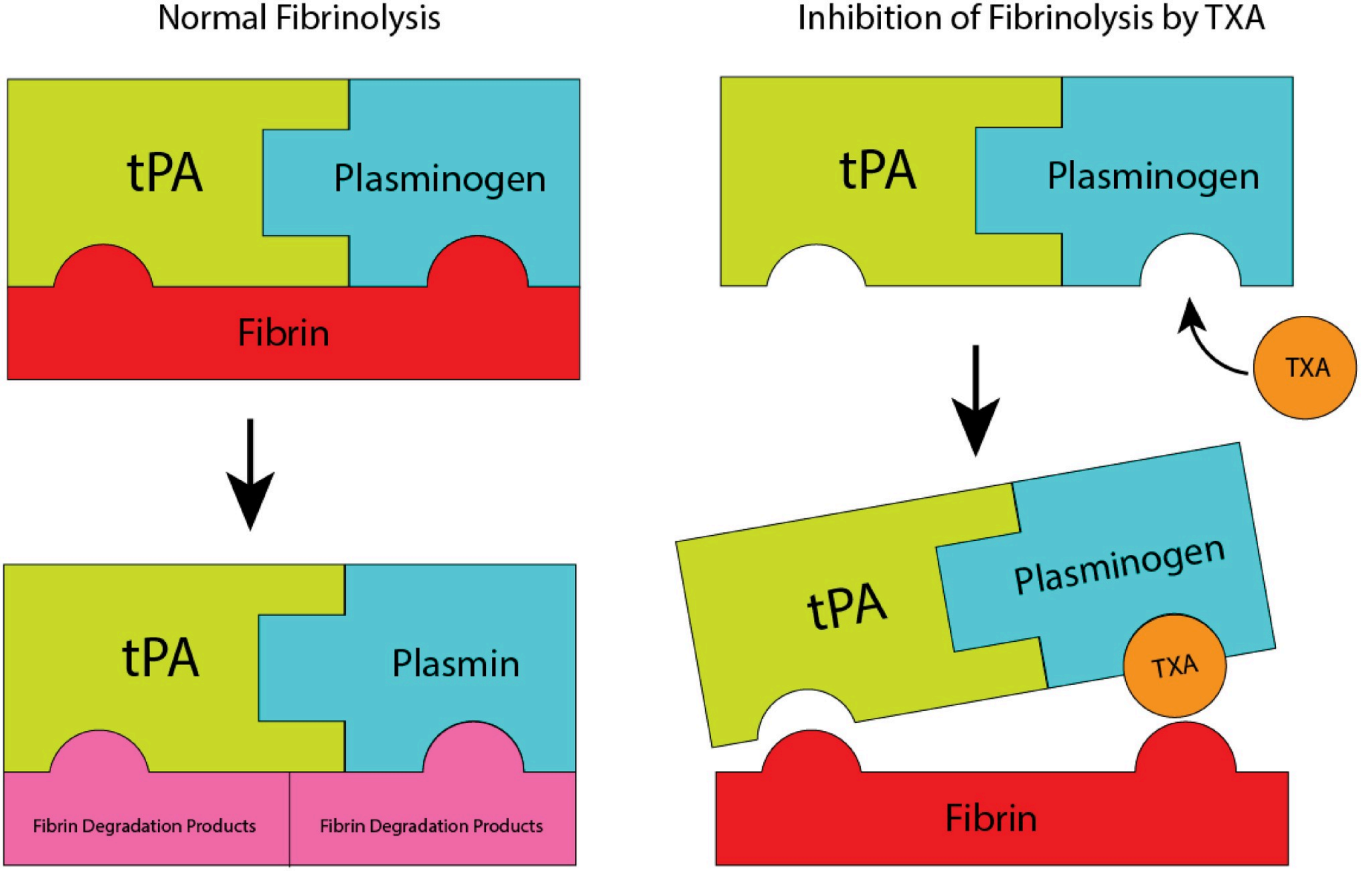
TXA interaction with plasminogen. Tranexamic Acid(TXA) inhibits fibrinolysis through its binding to plasminogen (Pg). This binding prevents plasminogen from binding to fibrin, which inhibits activation through tPA.

Tranexamic acid is an antifibrinolytic amino acid derivative that prevents the binding of plasmin(ogen) to fibrin. This binding inhibits fibrinolysis in two ways (Fig 1). First, it slows down tissue-plasminogen-activator(tPA) mediated conversion of plasminogen to plasmin, as fibrin acts as a substrate that increases the catalytic efficiency of the reaction 500-fold. Even after activation, TXA-bound plasmin cannot bind to fibrin, directly preventing the digestion of fibrin [7]. The mechanisms behind the anti-fibrinolytic properties of TXA are well understood, but the mechanism responsible for the possible increased bleeding and mortality have yet to be determined.

A possible explanation for the varying effectiveness of TXA treatment is the interaction between TXA and urokinase plasminogen activator (uPA or urokinase) [8]. Studies have shown that uPA levels are elevated in many instances in traumatic injury [9, 10], and since uPA-mediated plasmin activation occurs in solution, the inhibition of fibrin binding by TXA is inconsequential to this process. Furthermore, there is another binding site for TXA on plasminogen that induces a conformation change that speeds up plasmin activation approximately 3-fold [11]. However this explanation is incomplete, as multiple studies have shown that despite the increase of plasmin generation, TXA still inhibits fibrinolysis in uPA-mediated systems [12, 13]. This suggests that other conditions must be met to change the behavior of TXA from anti-fibrinolytic to pro-fibrinolytic. To explore the possible conditions under which TXA can increase fibrinolysis through uPA-mediated plasmin generation, we constructed a differential equation based computational model. This model builds on previous ODE models [14–18] of the coagulation and fibrinolysis system, but focuses specifically on investigating the interaction between uPA and TXA, which are not present in these models.

**Fig 2:**
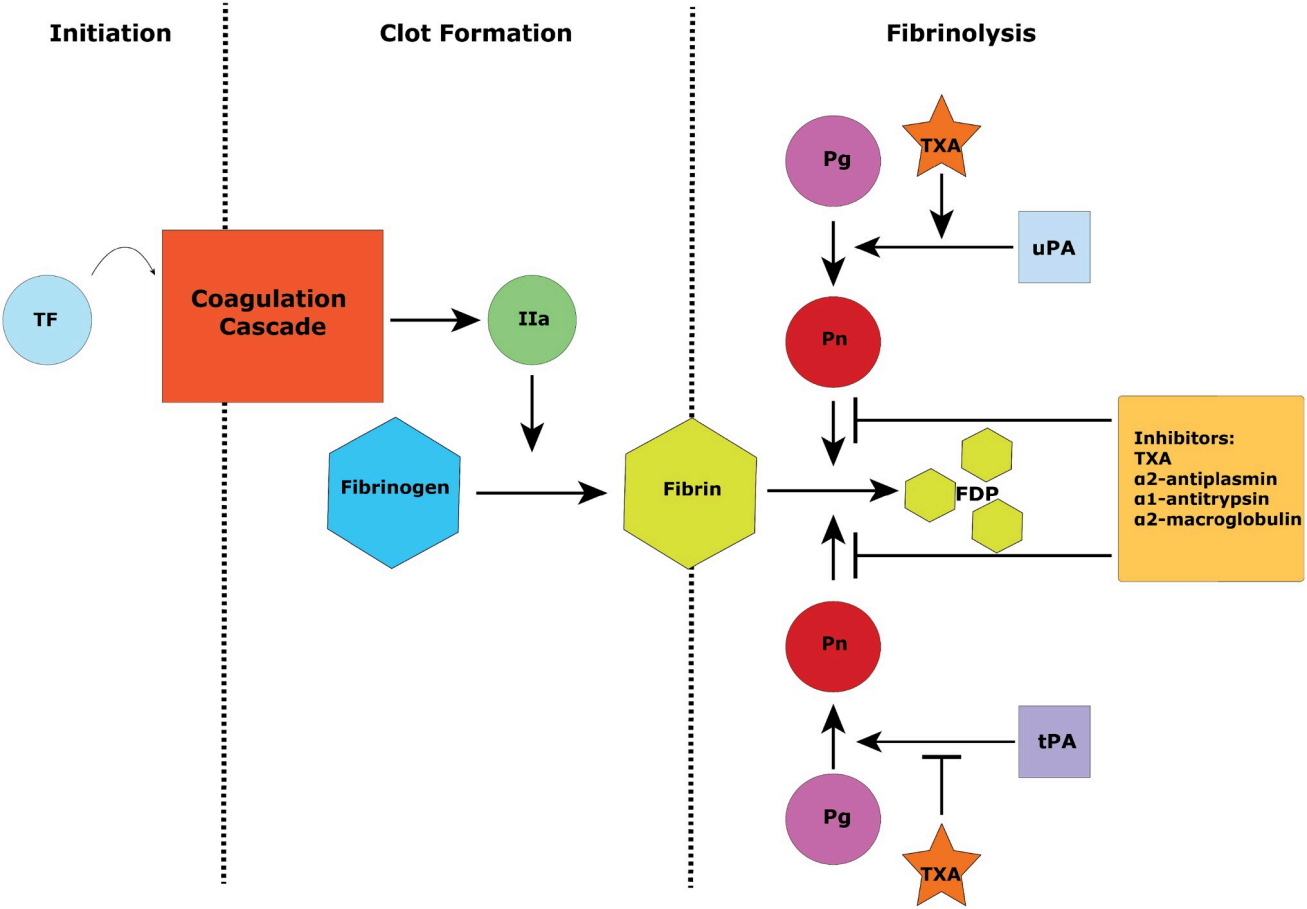
Simplified diagram of reaction network. This network diagram shows the important reactions in our model. Tissue factor (TF) initiates a chain of reactions in the coagulation cascade resulting in thrombin (IIa). Thrombin converts fibrinogen into fibrin, forming the clot. The clot is broken down by plasmin (Pn) which is activated from plasminogen (Pg) in one of two ways, by tissue-plasminogen activator (tPA) or urokinase-plasminogen activator (uPA). This diagram illustrates how tranexamic acid (TXA) has properties that both inhibit and promote fibrinolysis. The purpose of this paper is to explore which conditions cause the net effect to change from anti-fibrinolytic to pro-fibrinolytic. Additional abbreviation: Fibrin Degradation Productions (FDP).

We found that additional plasmin inhibitors such as *α*1-antitrypsin(A1AT) and *α*2-macroglobulin(A2M), two proteins not often associated with fibrinolysis, play a critical role in preventing TXA-induced hyperfibrinolysis (Fig 2. If the other plasmin inhibitors are depleted, *α*2-antiplasmin (AP or antiplasmin) is not sufficient in preventing fibrinolysis, and the presence of TXA acts to increase the rate of fibrinolysis.

## Materials and methods

### Computational model

In this paper we constructed an ordinary differential equation (ODE) model of the coagulation process. This includes reactions for thrombin generation, fibrin formation and fibrin degradation (Fig 2). Most of these reactions and their associated rate constants are taken from models based on in vitro experimental results, and most of them can be found in previously published ODE models [14–18]. In our model we also make the assumption that A2M and A1AT are not being consumed outside of the coagulation and fibrinolysis systems. Because of the difficulty of accurately modeling these additional systems and their low impact on the overall system, we took the conservative approach of excluding them. If these reactions were included, the rate of A2M and A1AT consumption would increase and the results would be amplified in the favor of our conclusions.

**Fig 3:**
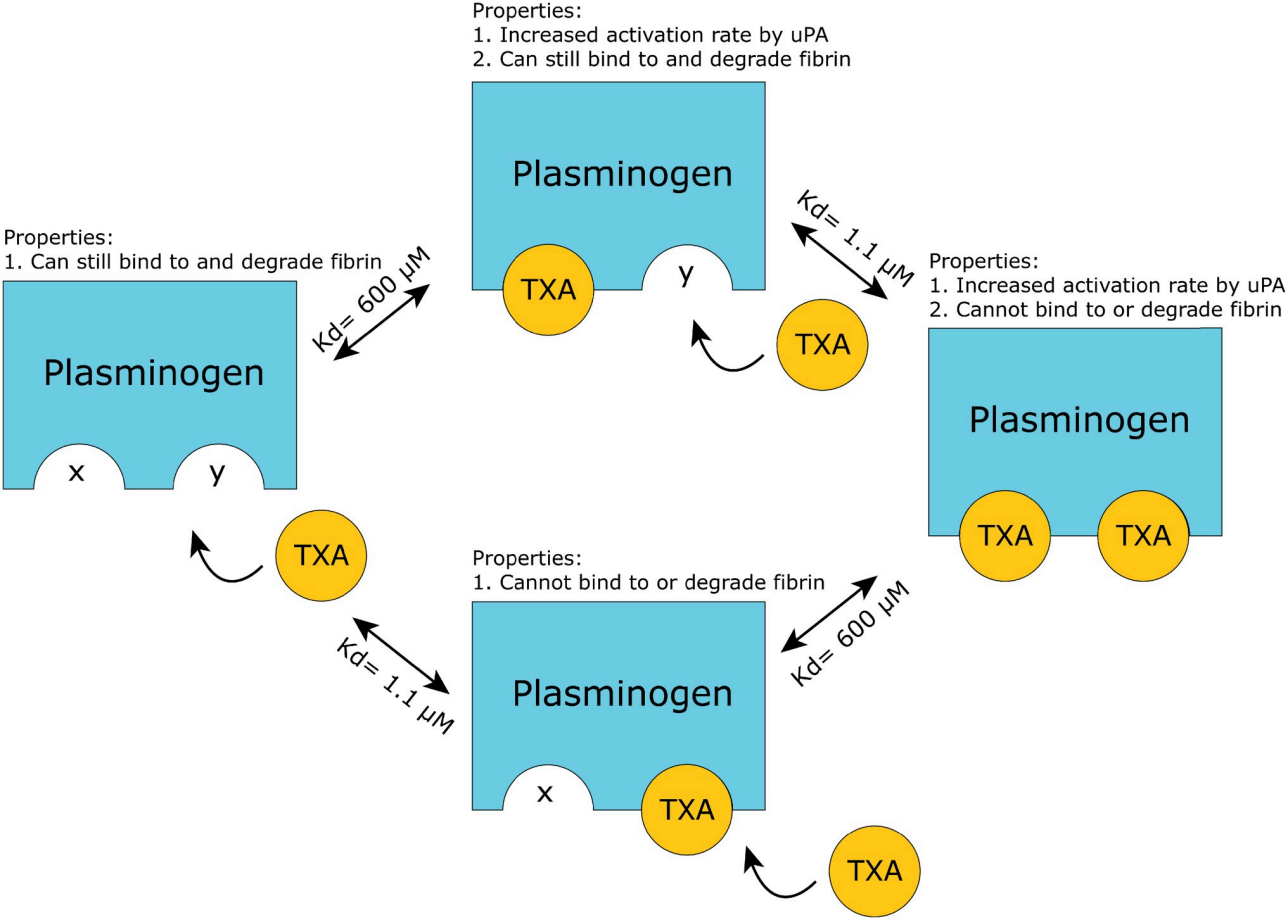
Diagram of different variants of TXA-bound plasminogen. Plasminogen has 2 binding sites that interact with TXA (labeled x and y in the model). One of the binding sites with *K*_*d*_ = 600 *μ*M changes the conformation that speeds up activation by uPA. The other binding site with *K*_*d*_ = 1.1 *μ*M prevents binding to fibrin, which prevents activation via tPA as well as fibrin degradation. UPA can activate all 4 of these variants, whereas tPA can activate only the variants with the second binding site free.

Our model focuses on the interactions between TXA and fibrinolysis, so it includes a more detailed model of TXA-plasminogen binding (Fig 3). We model plasminogen with 2 binding sites for TXA (labeled x and y), resulting in 4 species of Pg, depending on whether TXA is bound to one or both of those sites (Pg, Pgx, Pgy, Pgxy). The x site controls the pro-fibrinolytic activity of TXA. If TXA is bound to this site (*K*_*d*_ = 600 *μ*M), then its activation rate via uPA is increased 3-fold. The y site controls the anti-fibrinolytic activity of TXA and has a much higher binding affinity (*K*_*d*_ = 1.1 *μ*M). This is the binding site that fibrin and plasmin inhibitors such as *α*2-antiplasmin bind to. If the y site is occupied by TXA, it cannot bind to fibrin, which prevents it from being activated by tPA and prevents it from degrading fibrin even after being converted to plasmin. While tPA cannot activate the plasminogen species with its y site bound (Pgy, Pgxy), uPA can activate all of these species of plasminogen. Therefore, in uPA-mediated systems, TXA will increase the amount of plasmin generated [11, 12].

The reversibility of the TXA binding with plasmin(ogen) has an important consequence. It means that eventually TXA bound to the y site will be replaced by a irreversible plasmin inhibitor as long as there is some left in the system. This means that TXA is effective at inhibiting fibrinolysis only if there is a sufficient supply of plasmin inhibitors [18]. A complete description of the model, a complete list of reactions, rate constants and initial conditions for our model can be found in S1 Appendix in S1 File. The full model can be found online at https://github.com/taicheeze/coag_ode_julia.

We initiated coagulation with 5 pM of tissue factor (TF), which is frequently used in other computational models [15]. We examined the rate of fibrinolysis initiated with 2.5 nM tPA or 5 nM uPA, and varied the amount of TXA in the system (0, 1, 3, 14, 54 and 3470 *μ*M) to compare to experimental results to those of Longstaff 2019 [13].

## Results

### TPA mediated fibrinolysis

**Fig 4:**
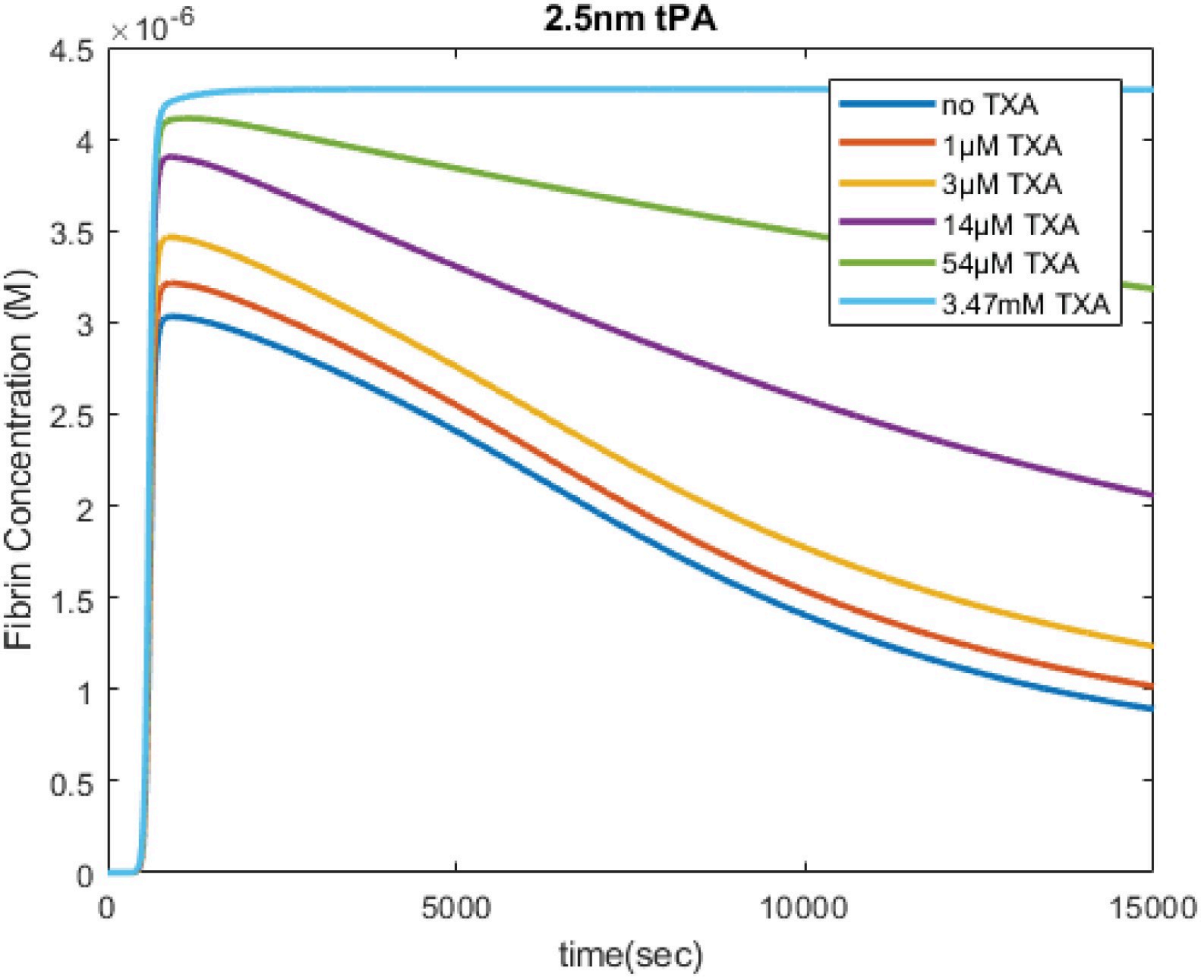
Effects of TXA on tPA mediated fibrinolysis. Modeling results show that Tranexamic Acid (TXA) affects tPA mediated fibrinolysis in a dose dependent fashion. As the concentration of TXA increases, the rate of fibrinolysis decreases. At the highest dose of TXA, fibrinolysis appears to be completely inhibited. These simulations are initiated with 5 pM tissue factor, and an initial tPA concentration of 2.5 nM.

The effects of TXA on tPA mediated fibrinolysis in our computational model behave as expected and are confirmed by results of previous empirical experiments(Fig 4) [12, 13, 19]. As more TXA was added to the system, fibrinolysis slowed down in a dose-dependent manner. This behavior is consistent through different initial concentrations of plasmin inhibitors (not shown) as expected since the interactions between TXA and tPA are straightforward and well established. These results show that the bulk of the model, with the exception of the uPA pathway of fibrinolysis, is in agreement with empirical data.

### UPA mediated fibrinolysis

**Fig 5:**
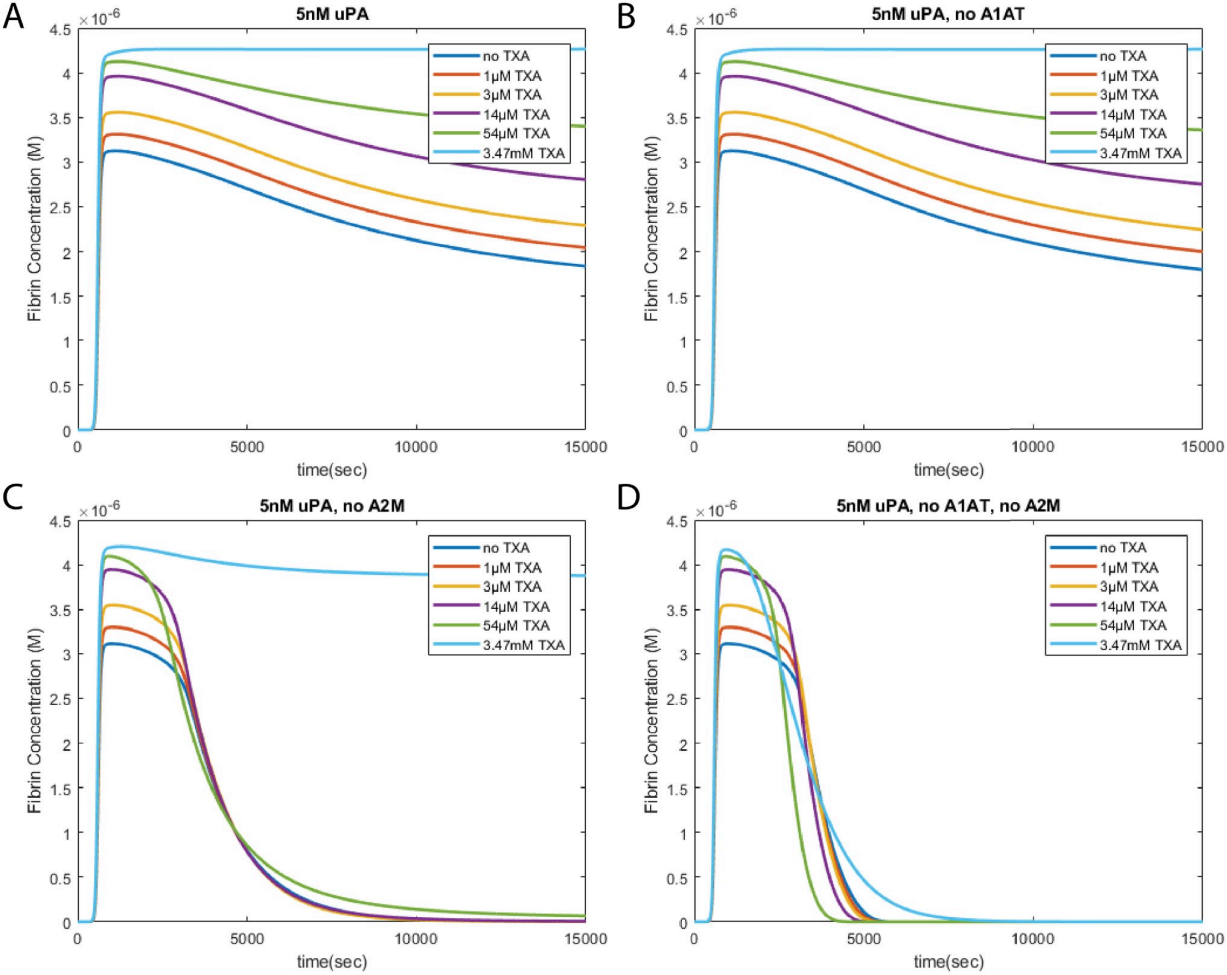
Effects of TXA on uPA mediated fibrinolysis. Modeling results reveal that the effect of TXA on uPA mediated fibrinolysis greatly depends on the presence or absence of plasmin inhibitors in the system. When A2M is in the system, with (A) or without (B) A1AT, fibrinolysis is inhibited and TXA behaves in an anti-fibrinolytic fashion. When A2M is removed from the system, but A1AT is still present (C), fibrinolysis can be observed. Under these circumstances, TXA exhibits anti-fibrinolytic behavior and acts to slow down fibrinolysis in a dose dependent manner. When both A2M and A1AT are removed from the system (D), TXA becomes pro-fibrinolytic, and speeds up the rate of fibrinolysis through increased plasmin generation and antiplasmin depletion. These simulations are initiated with 5 pM tissue factor, and an initial uPA concentration of 5 nM.

The interactions between TXA and uPA are more complex. Overall, fibrinolysis with uPA is slower than with tPA, which can be for a variety of reasons. UPA activates plasmin in solution rather than on the fibrin surface, making it easier to inhibit by plasmin inhibitors. In addition, uPA is inhibited by protein C inhibitor (PCI) [20, 21] which is present in the body at a higher concentration than the inhibitors of tPA. Since we are assuming that A2M and A1AT are not being consumed by reactions outside of the coagulation and fibrinolysis systems, this affects uPA-activated plasmin more than tPA activated plasmin. Focusing on the plasmin inhibition role of these proteins, we simulated the effects of consumption of these proteins by varying their initial concentrations. Under conditions simulated with high concentrations of plasmin inhibitors (Fig 5A), TXA behaves in an anti-fibrinolytic manner. Despite increasing the amount of plasmin in the system, the activated plasmin is quickly bound to TXA, preventing it from degrading fibrin. The TXA bound to the active plasmin is later replaced by one of the non-reversible plasmin inhibitors, which permanently inhibits it. Higher concentrations of TXA shift the equilibrium between TXA-bound plasmin and free plasmin, lowering the amount of free plasmin at any given time, resulting in quick inhibition by other plasmin inhibitors.

When *α*1-antitrypsin is removed from the system (Fig 5B), the behavior of the system remains mostly unchanged. The effects of A1AT are not obvious until both A1AT and A2M are removed. In this system, A1AT appears to play a role redundant to A2M except much weaker, due to its much slower binding to plasmin [22]. When A2M is removed from the system (Fig 5C), uPA mediated fibrinolysis becomes much more apparent. However, TXA retains its anti-fibrinolytic behavior, with a small change in behavior in the 10-100 *μ*M range, which has also been shown experimentally [12].

However, once both A2M and A1AT are removed from the system(Fig 5D), the behavior of TXA flips. In this system, the only plasmin inhibitor left is antiplasmin. Since the ratio of plasminogen to antiplasmin is nominally 2:1, a large amount of plasmin activation can deplete antiplasmin. The presence of TXA increases the rate of plasmin activation, and since the binding between TXA and plasmin is reversible, antiplasmin will eventually deplete, and any remaining activated plasmin will rapidly degrade fibrin, resulting in hyperfibrinolysis. Additional results of uPA mediated fibrinolysis with varying levels of plasmin inhibitors can be found in S1 Fig in the S1 File.

### TPA and uPA mediated fibrinolysis

**Fig 6:**
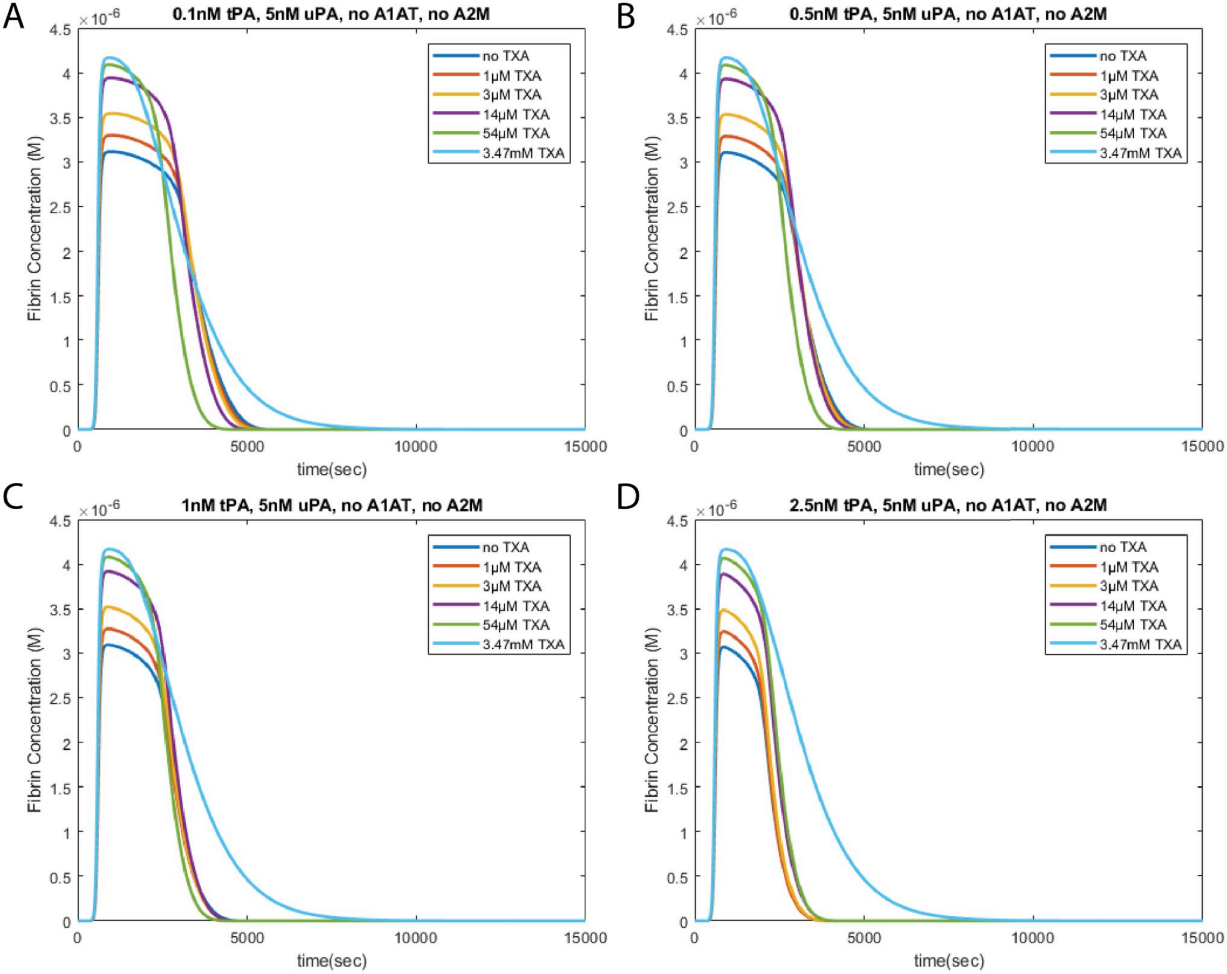
Effects of TXA on systems with both tPA and uPA. Modeling results show that the effect of TXA on systems with both tPA and uPA depend on the balance between the two pathways. The concentration of uPA is held constant at 5nM, as tPA is increased from 0.1nM to 2.5nM. When tPA is low compared tp uPA (A and B), TXA exhibits the pro-fibrinolytic behavior similar to Fig 5D. This behavior shifts toward anti-fibrinolytic as tPA increases. At 1nM tPA (C), the hyperfibrinolysis occurs at similar times regardless of the concentration of TXA. At 2.5nM tPA (D), TXA is anti-fibrinolytic and acts to slow down hyperfibrinolysis. These simulations are initiated with 5 pM tissue factor.

The behavior of TXA also depends on the balance between tPA and uPA in the system. In systems depleted of A1AT and A2M, TXA remains anti-fibrinolytic in the tPA pathway, but becomes pro-fibrinolytic in the uPA pathway. By modulating the ratio of tPA to uPA, the overall effect of TXA can go from pro-fibrinolytic (Fig 6A and 6B), to no effect(Fig 6C) to anti-fibrinolytic(Fig 6D). We can see that across these concentrations of tPA, the curves for the highest TXA concentrations are more-or-less identical. This shows that at high concentrations of TXA, the tPA pathway is completely inhibited.

## Discussion

In this paper we have proposed a mechanistic explanation for the variance of outcomes in patients treated with TXA. In situations with elevated uPA and depletion of plasmin inhibitors such as A1AT and A2M, TXA can speed up fibrinolysis, leading to increased bleeding. Without A1AT and A2M, antiplasmin is quickly depleted and the generated plasmin rapidly breaks down any fibrin clots uninhibited. One possible mechanism for depletion of plasmin inhibitors during trauma may arise from the interactions between A1AT and A2M with activated Protein C (APC) [23]. Many studies have shown that high levels of protein C are correlated with trauma patients with worse outcomes [24, 25]. This is more likely to occur in patients that arrive late, as there is more time for the plasmin inhibitors to deplete. Furthermore, the likely concurrence of high tPA in the system during trauma can work in tandem to push a system toward hyperfibrinolysis [26]. Under these conditions, TXA increases the amount of plasmin produced by uPA in solution that rapidly consumes antiplasmin. Once antiplasmin has been reduced significantly, TXA loses its ability to prevent tPA-mediated fibrinolysis and any plasmin generated on fibrin surfaces will go uninhibited as A2M and A1AT cannot bind to fibrin-bound plasmin.

This model also provides an explanation for empirical studies that show how TXA can increase plasmin generation in uPA systems, but at the same time slow down fibrinolysis. This model shows that TXA binding to plasmin alone cannot prevent antiplasmin from depleting, because the binding between TXA and plasmin is reversible. Regardless of the amount of TXA in the system, free plasmin will always be present, but higher concentrations of TXA decreases the amount of free plasmin available. This free plasmin will be consumed by plasmin inhibitors and shifts the equilibrium to generate more free plasmin. In healthy plasma, there is an ample supply of plasmin inhibitors, and TXA serves to lower the amount of free plasmin at any given time, leading to quick inhibition. However, if A2M and A1AT are depleted, TXA can act to speed up the rate of antiplasmin depletion because the concentration of plasminogen is approximately twice the concentration of antiplasmin. This leaves the remaining free plasmin free to break down fibrin unimpeded. Because of this, the presence of additional irreversible inhibitors is necessary in a functional fibrinolytic system.

Even in the absence of TXA, these additional inhibitors are necessary in healthy fibrinolysis. During an injury, tPA deposits in endothelial cells are quickly released in large amounts into the site of injury. During the time immediately after injury, plasmin activators greatly outnumber its inhibitors such as PAI-1 and can quickly activate large amounts of plasmin [18]. Without additional inhibitors, antiplasmin would quickly deplete, resulting in hyperfibrinolysis.

As TXA continues to be explored as treatment for different situations [27–29], a mechanistic understanding of this phenomena becomes increasingly important in preventing unforeseen side effects of TXA treatment. Currently, the decision on whether to use TXA in treatment depends on whether the patient arrived before or after 3 hours post injury. Although this heuristic protocol can be helpful, it will undoubtedly lead to instances in which patients who can benefit from TXA treatment are not receiving it and patients who would suffer adversely from it will be given it. With an understanding of the conditions under which TXA helps or worsens a patient’s condition, we can identify patients that should receive TXA and ones that shouldn’t. In addition, TXA treatment can potentially be improved by using plasmin inhibitors in conjection with TXA to mitigate the risk of hyperfibrinolysis through the depletion of plasmin inhibitors.

## Conclusion

This paper uses computational modeling to present a mechanistic explanation for the different behaviors of tranexamic acid. TXA usually has anti-fibrinolytic properties, but in systems with high levels of uPA and low levels of plasmin inhibitors, TXA can become pro-fibrinolytic, resulting in worse outcomes. This understanding can potentially be used to adjust treatment protocols and stratify patients according to TXA risk to ensure that treatment is beneficial to patients receiving it. As we have done with previous models [18], this model can also be used to test potential treatments such as the replenishment of key proteins such as *α*2-antiplasmin.

## Supporting information

S1 File (PDF)
