# Supplementary material for: Computational model of tranexamic acid on urokinase mediated fibrinolysis
Source: PLoS One. 2020 May 26;15(5):e0233640. doi: 10.1371/journal.pone.0233640 (PMC7250412; doi:10.1371/journal.pone.0233640)
Supplement: S1 File — (PDF) [file pone.0233640.s001.pdf]

S1 Appendix

Model Details

Abbreviations

Roman numerals designate the coagulation factors, and if followed by a lower case letter a, it indicates the activated version. Exceptions to this are II(prothrombin), IIa(thrombin), mIIa(meizothrombin). Va3, Va5, Va53 represent partially proteolized forms of factor Va. A underscore between species names indicate a complex formed between those species. Other abbreviations are as follows: AP, α_2-antiplasmin; APC, activated protein C; ATIII, antithrombin-III; FDP, fibrin degradation products; Fg, fibrinogen; FnI, fibrin I monomers; FnII, fibrin II monomers; FPA, fibrinopeptide A; FPB, fibrinopeptide B; HCF and LCA1, factor Va degradation products; PAI, plasminogen activator inhibitor-1; PC, protein C; Pg, plasminogen; Pn, plasmin; TF, tissue factor; TFPI, tissue factor pathway inhibitor; TM, thrombomodulin, stPA, soluble tissue-plasminogen activator; uPA, urokinase plasminogen activator; TXA, tranexamic acid; PCI, protein c inhibitor; a2mg, α2-macroglobulin; a1at, α1-antitrypsin. Pgx, Pgy, Pgxy, Pnx, Pnz, Pnxz refer to plasminogen or plasmin bound to TXA at specific binding sites. APnc refers to antiplasmin without the c-terminal region.

S1 Table

List of Reactions

#	Reaction	$k_{forward}(1/M*s)^*$	$k_{reverse}(1/s)^*$
1	TF + VII ⇌ TF_VII	3.20E+06	3.10E-03
2	TF + VIIa ⇌ TF_VIIa	2.30E+07	3.10E-03
3	TF_VIIa + VII → TF_VIIa + VIIa	4.40E+05†	
4	Xa + VII → Xa + VIIa	1.30E+07	
5	Iia + VII → Iia + VIIa	2.30E+04	
6	TF_VIIa + X ⇌ TF_VIIa_X	2.50E+07	1.05E+00
7	TF_VIIa_X → TF_VIIa_Xa	6.00E+00†	
8	TF_VIIa + Xa ⇌ TF_VIIa_Xa	2.20E+07	1.90E+01
9	TF_VIIa + IX ⇌ TF_VIIa_IX	1.00E+07	2.40E+00
10	TF_VIIa_IX → TF_VIIa + Ixa	1.80E+00†	
11	Xa + II → Xa + Iia	7.50E+03	
12	Iia + VIII → Iia + VIIIa	2.00E+07	
13	VIIIa + IXa ⇌ VIIIa_IXa	1.00E+07	5.00E-03
14	VIIIa_IXa + X ⇌ VIIIa_IXa_X	1.00E+08	1.00E-03
15	VIIIa_IXa_X → VIIIa_IXa + Xa	8.20E+00†	
16	VIIIa ⇌ VIIIlca1 + VIIIa2	6.00E-03	2.20E+04‡
17	VIIIa_IXa_X → VIIIlca1 +VIIIa2 + X + IXa	1.00E-03†	
18	VIIIa_IXa → VIIIlca1 +VIIIa2 + IXa	1.00E-03†	
19	IIa + V → IIa + Va	2.00E+07	
20	Va + Xa ⇌ Xa_Va	4.00E+08	2.00E-01
21	Xa_Va + II ⇌ Xa_Va_II	1.00E+08	1.03E+02
22	Xa_Va_II → Xa_Va + mIIa	6.35E+01†	
23	mIIa + Xa_Va → IIa + Xa_Va	2.30E+08	
24	Xa + TFPI ⇌ Xa_TFPI	9.00E+05	3.60E-04
25	TF_VIIa_Xa + TFPI ⇌ TF_VIIa_Xa_TFPI	3.20E+08	1.10E-04
26	TF_VIIa+Xa_TFPI → TF_VIIa_Xa_TFPI	5.00E+07	
27	Xa + ATIII → Xa_ATIII	4.20E+03	
28	mIIa + ATIII → mIIa_ATIII	7.10E+03	
29	IXa + ATIII → IXa_ATIII	4.90E+02	
30	IIa + ATIII → IIa_ATIII	1.60E+04	
31	TF_VIIa + ATIII → TF_VIIa_ATIII	2.30E+02	
32	TM + IIa ⇌ TM_IIa	1.00E+08	3.30E-01
33	TM_IIa + PC ⇌ TM_IIa_PC	1.00E+08	1.00E+02
34	TM_IIa_PC → TM_IIa + APC	4.10E-01	
35	TM_IIa + ATIII → TM_IIa_ATIII	7.10E+03	
36	APC + Va ⇌ APC_Va	1.00E+08	7.00E-01
37	APC_Va → APC + Va5	1.00E+00†	
38	APC_Va → APC +Va3	1.92E-01†	
39	APC + Va5 ⇌ APC_Va5	1.00E+08	7.00E-01
40	APC + Va5 ⇌ APC_Va5	1.00E+08	7.00E-01
41	APC_Va3 → APC + Va53	1.00E+00†	
42	APC_Va5 → APC + Va53	1.92E-01†	

*Default units unless specified.
†Units of s^{-1}.
‡Units of $1/M*s$.

S1 Table

List of Reactions cont.

#	Reaction	$k_{forward}(1/M*s)^*$	$k_{reverse}(1/s)^*$
43	Va3 → HCF + LCA1	2.80E-02†	
44	Va53 → HCF + LCA1	2.80E-02†	
45	APC+LCA1 ⇌ APC_LCA1	1.00E+08	7.00E-01
46	TM_IIa + APC ⇌ TM_IIa_APC	1.00E+08	1.00E+02
47	Va5 + Xa ⇌ Xa_Va5	1.50E+08	1.50E-01
48	Va3 + Xa ⇌ Xa_Va3	1.50E+08	1.50E-01
49	Xa_Va5 + II ⇌ Xa_Va5_II	1.00E+08	1.03E+02
50	Xa_Va5_II → Xa_Va5 + mIIa	1.03E+01†	
51	Xa_Va3 + II ⇌ Xa_Va3_II	1.00E+08	1.03E+02
52	Xa_Va3_II → Xa_Va3 + mIIa	1.03E+01†	
53	mIIa + Xa_Va5 → IIa + Xa_Va5	4.60E+07	
54	mIIa + Xa_Va3 → IIa + Xa_Va3	4.60E+07	
55	Xa_Va3 → Xa + HCF + LCA1	3.50E-03†	
56	Xa_Va3_II → Xa + II + HCF + LCA1	3.50E-03†	
57	IXa + X → IXa + Xa	5.70E+03	
58	mIIa + V → mIIa + Va	3.00E+06	
59	TM + mIIa ⇌ TM_mIIa	1.00E+08	3.30E-01
60	TM_mIIa + PC ⇌ TM_mIIa_PC	1.00E+08	1.00E+02
61	TM_mIIa_PC → TM_mIIa + APC	4.10E-01†	
62	TM_mIIa + ATIII → TM_mIIa_ATIII	7.10E+03	
63	Va53 + Xa ⇌ Xa_Va53	1.50E+08	1.50E-01
64	Xa_Va53 + II ⇌ Xa_Va53_II	1.00E+08	1.03E+02
65	Xa_Va53_II → Xa_Va53 + mIIa	1.03E+01†	
66	mIIa + Xa_Va53 → IIa + Xa_Va53	4.60E+07	
67	Xa_Va53 → Xa + HCF + LCA1	3.50E-03†	
68	Xa_Va53_II → Xa + II + HCF + LCA1	3.50E-03†	
69	II + Va ⇌ II_Va	1.00E+08	7.00E+01
70	Xa_Va5 + APC → Xa_Va53 + APC	4.05E+06	
71	APC → NULL	1.10E-03†	
72	Fg + IIa ⇌ Fg_IIa	1.00E+08	7.20E+02
73	Fg_IIa → FnI + IIa + FPA	8.40E+01†	
74	FnI + IIa ⇌ FnI_IIa	1.00E+08	7.50E+02
75	FnI_IIa → FnII + IIa + FPB	7.40E+00†	
76	2FnI ⇌ FnI2	1.00E+06	6.40E-02
77	FnI2 + IIa ⇌ FnI2_IIa	1.00E+08	7.50E+02
78	FnI2_IIa ⇌ FnII2 + IIa + 2FPB	4.90E+01†	
79	FnII + IIa ⇌ FnII_IIa	1.00E+08	1.00E+03
80	FnI2_IIa + ATIII → FnI2_IIa_ATIII	1.60E+04	
81	FnI_IIa + ATIII → FnI_IIa_ATIII	1.60E+04	

*Default units unless specified.

†Units of s^{-1}.

S1 Table

List of Reactions cont.

#	Reaction	$k_{forward}(1/M*s)^*$	$k_{reverse}(1/s)^*$
82	FnII_IIa + ATIII → FnII_IIa_ATIII	1.00E+04	
83	Pn + AP → Pn_AP	3.00E+06	
84	stPA + FnII ⇌ stPA_FnII	1.00E+04	5.80E-03
85	stPA + FnII2 ⇌ stPA_FnII2	1.00E+04	5.80E-03
86	stPA + PAI → stPA_PAI	4.50E+05	
87	Pn_FnII → Pn + FDP	3.13E-01†	
88	Pn_FnII2 → Pn + 2FDP	3.13E-01†	
89	APC + PAI → APC_PAI	1.80E+05	
90	Pg → Pn (uPA)‡		
91	Pgx → Pgx (uPA)‡		
92	TXA + Pg ⇌ Pgx	1.00E+08	6.00E+04
93	PAI + uPA → uPA_PAI	7.90E+06	
94	Pn + TXA ⇌ Pnx	1.00E+08	6.00E+04
95	Pg + FnII ⇌ Pg_FnII	1.00E+05	2.20E-01
96	Pg + FnII2 ⇌ Pg_FnII2	1.00E+05	2.20E-01
97	Pg_FnII → Pn_FnII (tPA)§		
98	Pg_FnII2 → Pn_FnII2 (tPA)§		
99	Pn + FnII ⇌ Pn_FnII	1.00E+05	5.00E-02
100	Pn + FnII2 ⇌ Pn_FnII2	1.00E+05	5.00E-02
101	Pgx + TXA ⇌ Pgxy	1.00E+08	1.10E+02
102	Pg + TXA ⇌ Pgy	1.00E+08	1.10E+02
103	Pgy + TXA ⇌ Pgxy	1.00E+08	6.00E+04
104	Pgx + FnII2 ⇌ Pgx_FnII2	1.00E+05	2.20E+00
105	Pnx + FnII2 ⇌ Pnx_FnII2	1.00E+05	5.00E-02
106	Pgxy → Pnxz (uPA)‡		
107	Pn + TXA ⇌ Pnz	1.00E+08	4.50E+03
108	Pnx + TXA ⇌ Pnxz	1.00E+08	4.50E+03
109	Pnz + TXA ⇌ Pnxz	1.00E+08	6.00E+04
110	Pgy → Pn + TXA (uPA)‡		
111	Pgx → Pnx (tPA)§		

*Default units unless specified.

†Units of s^{-1}.

‡The reaction rate of uPA-mediated plasmin activation is determined using the following Michaelis-Menten formulation:

$$r_n = \frac{K_1[uPA][Pg]}{K_2+[Pg]}$$

where r_n is the reaction rate associated with reaction n, [Pg] is the plasminogen species in reaction n, $K_1 = 0.73s^{-1}$, for Pg, Pgy and $K_1 = 1.85s^{-1}$ for Pgx and Pgxy, $K_2 = 2.5x10^{-5}M$

§The reaction rate of tPA-mediated plasmin activation is determined using the following Michaelis-Menten formulation:

$$r_n = \frac{K_3[tPA][Pg]}{K_4+[Pg]}$$

where r_n is the reaction rate associated with reaction n, [Pg] is the plasminogen species in reaction n, $K_1 = 0.11s^{-1}$, $K_4 = 1.9x10^{-7}M$

S1 Table

List of Reactions cont.

#	Reaction	$k_{forward}(1/M*s)^*$	$k_{reverse}(1/s)^*$
112	Pnx_FnII2 → Pnx + 2FDP	3.13E-01†	
113	Pnx + AP → Pn_AP	7.50E+04	
114	Pnz + AP → Pn_AP	7.50E+04	
115	Pnxz + AP → Pn_AP	7.50E+04	
116	Pn_FnII2 + AP → Pn_AP + FnII2	7.50E+04	
117	Pnx_FnII2 + AP → Pn_AP +FnII2	7.50E+04	
118	Pg_FnII2 → Pn_FnII2 (uPA)‡		
119	Pgx_FnII2 → Pnx_FnII2 (uPA)‡		
120	uPA + PCI → NULL	2.00E+03	
121	Pn + APnc → Pn_AP	7.50E+04	
122	Pnx + APnc → Pn_AP	1.88E+03	
123	Pnx_FnII2 + APnc → Pn_AP	1.88E+03	
124	Pn + A2M → Pn_A2M	1.11E+05	
125	Pnx + A2M → Pn_A2M	1.11E+05	
126	Pnz + A2M → Pn_A2M	1.11E+05	
127	Pnxz + A2M → Pn_A2M	1.11E+05	
128	Pn + A1AT → Pn_A1AT	4.00E+02	
129	Pnx + A1AT → Pn_A1AT	4.00E+02	
130	Pnz + A1AT → Pn_A1AT	4.00E+02	
131	Pnxz + A1AT → Pn_A1AT	4.00E+02	

Reactions and rate constants for reactions 1-71 come from Ref. (3). Reactions and rate constants for reactions 72-82 come from analysis on Ref.(13) done by Ref.(12). Reaction 83 and the corresponding rate constant come from Ref.(15). Reactions and reaction rates for reactions 84-85, 95-96, 99-100, 104-105 come from Ref.(18). Reaction 86 and the corresponding rate constant come from Ref.(7). Reactions and reaction rates for reactions 87, 88, 112 come from Ref.(9). Reaction 89 and the corresponding rate constant come from Ref.(8). Reactions and reaction rates for reactions 91, 93, 106, 110, 118-119 come from Ref.(5). Reactions and reaction rates for reactions 92, 94, 101-103, 107-109 come from Ref.(4). Reaction 97, 98, 111 and the corresponding kinetic constants come from Ref.(19). Reactions and reaction rates for reactions 112-117, 121-123 come from Ref.(1). Reaction 120 and the corresponding rate constant come from Ref.(16). Reactions and reaction rates for reactions 124-131 come from Ref.(10).

*Default units unless specified.

†Units of s^{-1}.

‡The reaction rate of uPA-mediated plasmin activation is determined using the following Michaelis-Menten formulation:

$$r_n = \frac{K_1[uPA][Pg]}{K_2+[Pg]}$$

where r_n is the reaction rate associated with reaction n, [Pg] is the plasminogen species in reaction n, $K_1 = 0.73s^{-1}$, for Pg, Pgy and $K_1 = 1.85s^{-1}$ for Pgx and Pgxy, $K_2 = 2.5x10^{-5}M$

S2 Table

Initial Values

Species*	Initial Value†	Reference
VII	1.00E-08	(3)
TF	5.00E-12	(3)
VIIa	1.00E-10	(3)
X	1.60E-07	(3)
IX	9.00E-08	(3)
II	1.60E-06	(3)
VIII	7.00E-10	(3)
V	2.00E-08	(3)
TFPI	2.50E-09	(3)
ATIII	3.40E-06	(3)
TM	1.00E-09	(3)
PC	6.50E-08	(3)
FG	9.00E-06	(2)
AP	6.50E-07	(14)
stPA	varies	(17)
PAI	5.00E-11	(17)
Pg	2.00E-06	(14)
uPA	0, 5e-9	
TXA	varies	
PCI	9.00E-08	(6)
APnc	3.50E-07	(1)
A2M	0, 3.4e-6	(11)
A1AT	0, 3.5e-5	(11)

*If unlisted, initial value is zero

†Units are in M

S1 Figure

The effect of TXA on various compositions of plasmin inhibitors.

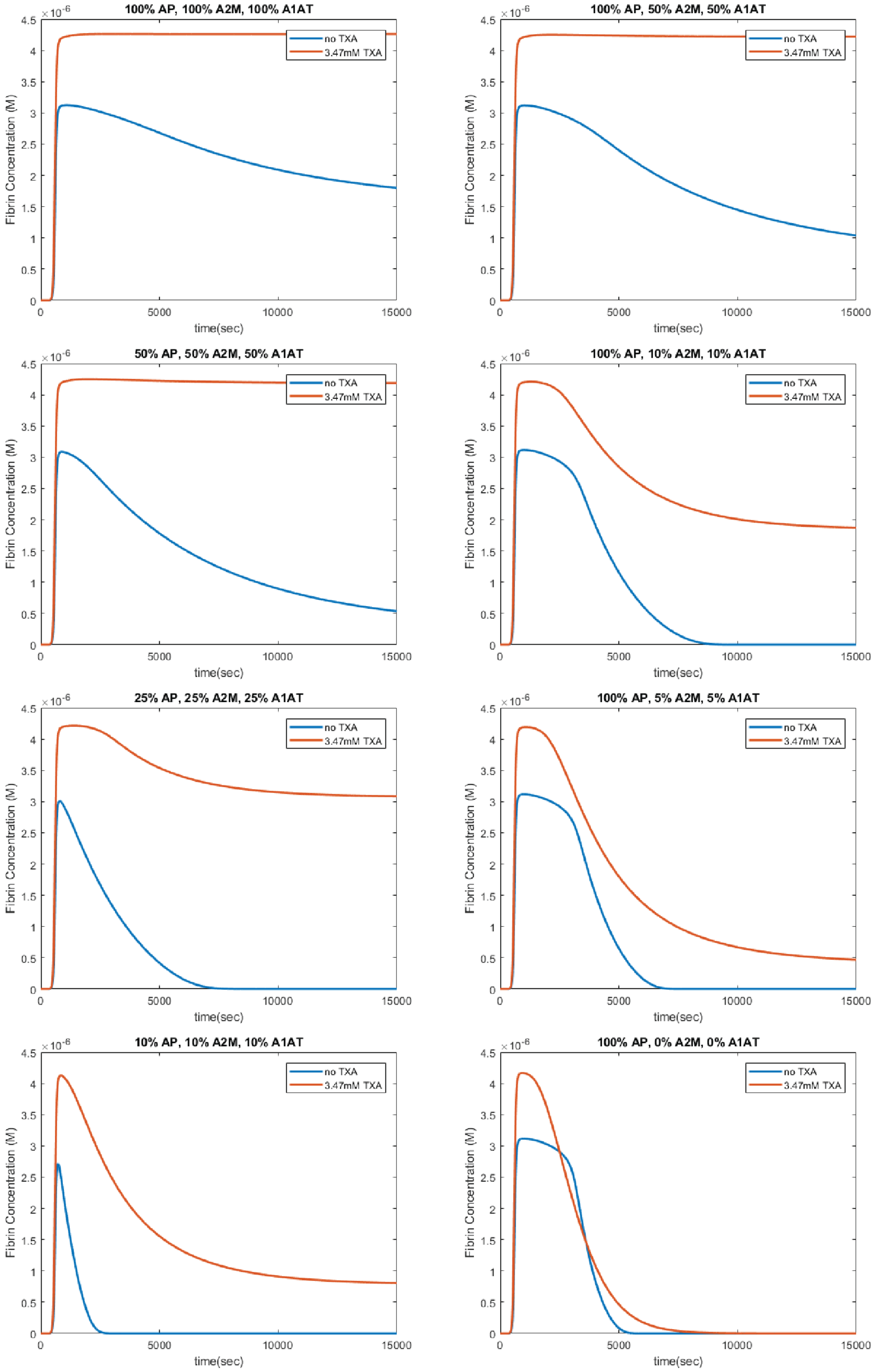

Figure S1: This figure shows the behavior of the fibrinolytic system with various initial concentrations of plasmin inhibitors. Lower levels of α2-antiplasmin has a strong effect with and without TXA whereas A2M and A1AT have a much more pronounced effect in the systems with TXA.

Supporting References

[1] Abdul, S., F. W. Leebeek, D. C. Rijken, and S. U. De Willige. Natural heterogeneity of α2-antiplasmin: Functional and clinical consequences. Blood 127:538–545, 2016.

[2] Brummel, K. E., S. Butenas, and K. G. Mann. An integrated study of fibrinogen during blood coagulation 6. J Biol.Chem. 274:22862–22870, 1999.

[3] Brummel-Ziedins, K. E., T. Orfeo, P. W. Callas, M. Gissel, K. G. Mann, and E. G. Bovill. The prothrombotic phenotypes in familial protein C deficiency are differentiated by computational modeling of thrombin generation. PLoS One 7:e44378, 2012.

[4] Castellino, F. J. and J. R. Powell. [29] Human plasminogen. pp. 365–378. 1981.

[5] Ellis, V., N. Behrendt, and K. Dano. Plasminogen activation by receptor-bound urokinase: A kinetic study with both cell-associated and isolated receptor. Journal of Biological Chemistry 266:12752–12758, 1991.

[6] Heeb, M. J., F. España, M. Geiger, D. Collen, D. C. Stump, and J. H. Griffin. Immunological identity of heparin-dependent plasma and urinary protein C inhibitor and plasminogen activator inhibitor-3. The Journal of biological chemistry 262:15813–6, 1987.

[7] Hekman Loskutoff, D. J., C. M. Kinetic Analysis of the Interactions between Plasminogen Activator Inhibitor 1 and both Urokinase and Tissue Plasminogen Activator. Arch. Biochem. Biophys. 262:199–210, 1988.

[8] Horrevoets, A. J. G. Plasminogen activator inhibitor 1 (PAI-1): In vitro activities and clinical relevance. British Journal of Haematology 125:12–23, 2004.

[9] Kim, P. Y., R. J. Stewart, S. M. Lipson, and M. E. Nesheim. The relative kinetics of clotting and lysis provide a biochemical rationale for the correlation between elevated fibrinogen and cardiovascular disease. J. Thromb. Haemost. 5:1250–1256, 2007.

[10] Kolev, K., I. Léránt, K. Tenekejiev, and R. Machovich. Regulation of fibrinolytic activity of neutrophil leukocyte elastase, plasmin, and miniplasmin by plasma protease inhibitors. The Journal of biological chemistry 269:17030–4, 1994.

[11] Levi, M., D. Roem, A. Kamp, J. de Boer, C. Hack, and J. ten Cate. Assessment of the Relative Contribution of Different Protease Inhibitors to the Inhibition of Plasmin In Vivo. Thrombosis and Haemostasis 69:141–146, 1993.

[12] Mitrophanov, A. Y., A. S. Wolberg, and J. Reifman. Kinetic model facilitates analysis of fibrin generation and its modulation by clotting factors: implications for hemostasis-enhancing therapies. Mol. Biosyst. 10:2347, 2014.

[13] Naski, M. C. and J. a. Shafer. A kinetic model for the alpha-thrombin-catalyzed conversion of plasma levels of fibrinogen to fibrin in the presence of antithrombin III. J. Biol. Chem. 266:13003–10, 1991.

[14] Rijken, D. C. and H. R. Lijnen. New insights into the molecular mechanisms of the fibrinolytic system. J. Thromb. Haemost. 7:4–13, 2009.

[15] Schneider, M., N. Brufatto, E. Neill, and M. Nesheim. Activated Thrombin-activatable Fibrinolysis Inhibitor Reduces the Ability of High Molecular Weight Fibrin Degradation Products to Protect Plasmin from Antiplasmin. J. Biol. Chem. 279:13340–13345, 2004.

[16] Stump, D. C., M. Thienpont, and D. Collen. Purification and characterization of a novel inhibitor of urokinase from human urine. Quantitation and preliminary characterization in plasma. The Journal of biological chemistry 261:12759–66, 1986.

[17] van Hinsbergh VWM. Regulation of the synthesis and secretion of plasminogen activators by endothelial cells, volume 18. 1988, 307–327 pp.

[18] Wootton, D. M., A. S. Popel, and B. Rita Alevriadou. An experimental and theoretical study on the dissolution of mural fibrin clots by tissue-type plasminogen activator. Biotechnology and Bioengineering 77:405–419, 2002.

[19] Zamarron, C., H. R. Lijnen, and D. Collen. Kinetics of the activation of plasminogen by natural and recombinant tissue-type plasminogen activator. J. Biol. Chem. 259:2080–2083, 1984.
